# An Integrated *Drosophila* Model System Reveals Unique Properties for F14512, a Novel Polyamine-Containing Anticancer Drug That Targets Topoisomerase II

**DOI:** 10.1371/journal.pone.0023597

**Published:** 2011-08-10

**Authors:** Sonia Chelouah, Caroline Monod-Wissler, Christian Bailly, Jean-Marc Barret, Nicolas Guilbaud, Stéphane Vispé, Emmanuel Käs

**Affiliations:** 1 Université de Toulouse, UPS, Université Paul Sabatier, Laboratoire de Biologie Moléculaire Eucaryote; Toulouse; France; 2 CNRS, Centre National de la Recherche Scientifique, UMR5099, Laboratoire de Biologie Moléculaire Eucaryote, Toulouse, France; 3 Centre de Recherche en Oncologie Expérimentale, Institut de Recherche Pierre Fabre, Toulouse, France; Institut Pasteur, France

## Abstract

F14512 is a novel anti-tumor molecule based on an epipodophyllotoxin core coupled to a cancer-cell vectoring spermine moiety. This polyamine linkage is assumed to ensure the preferential uptake of F14512 by cancer cells, strong interaction with DNA and potent inhibition of topoisomerase II (Topo II). The antitumor activity of F14512 in human tumor models is significantly higher than that of other epipodophyllotoxins in spite of a lower induction of DNA breakage. Hence, the demonstrated superiority of F14512 over other Topo II poisons might not result solely from its preferential uptake by cancer cells, but could also be due to unique effects on Topo II interactions with DNA. To further dissect the mechanism of action of F14512, we used *Drosophila melanogaster* mutants whose genetic background leads to an easily scored phenotype that is sensitive to changes in Topo II activity and/or localization. F14512 has antiproliferative properties in *Drosophila* cells and stabilizes ternary Topo II/DNA cleavable complexes at unique sites located in moderately repeated sequences, suggesting that the drug specifically targets a select and limited subset of genomic sequences. Feeding F14512 to developing mutant *Drosophila* larvae led to the recovery of flies expressing a striking phenotype, "*Eye wide shut*," where one eye is replaced by a first thoracic segment. Other recovered F14512-induced gain- and loss-of-function phenotypes similarly correspond to precise genetic dysfunctions. These complex *in vivo* results obtained in a whole developing organism can be reconciled with known genetic anomalies and constitute a remarkable instance of specific alterations of gene expression by ingestion of a drug. "*Drosophila*-based anticancer pharmacology" hence reveals unique properties for F14512, demonstrating the usefulness of an assay system that provides a low-cost, rapid and effective complement to mammalian models and permits the elucidation of fundamental mechanisms of action of candidate drugs of therapeutic interest in humans.

## Introduction

Topoisomerase II (Topo II) is an essential enzyme that handles topological reactions in all living cells and is required for different aspects of DNA metabolism, from the resolution of constraints resulting from packaging of DNA into chromatin, to its transcription and replication and, most importantly, the condensation [1] and segregation of sister chromatids in the course of cell division (reviewed in [2], [3], [4]). As such, it constitutes a target of choice for anticancer drugs ([5], [6], [7] and references therein). Because the basic Topo II-mediated reaction is central to such key aspects of cellular activities, drugs that target its catalytic cycle and, most commonly, poison it, have been extensively used in anti-cancer therapies [8]. These include etoposide (VP16) and teniposide (VM26) still being widely used in human [6], [9]. However, widespread applications have been limited by their high cytotoxicity to normal cells as well as the induction of drug resistance [10], [11].

In proliferating cells, Topo II is an abundant enzyme, with approximately 2–3×10^5^ molecules/nucleus [1] preferentially localized at sites found every 50–80 kbp [12]. Treatment of cells with Topo II poisons such as etoposide or teniposide can result in the formation of meta-stable “cleavable complexes” [13] located 30–50 kpb apart that can be converted to DNA double-stranded breaks as a result of collisions with replication or transcription machineries. Collisions with any DNA motor complex can result in the conversion of the readily reversible cleavable complex into an irreversible cytotoxic lesion. Accumulation of these lesions, which cannot be easily handled by the cellular DNA repair machinery, usually induces apoptotic pathways that result in the onset of cell death within approximately 48–72 hours in the case of dividing cells [14], [15], [16], [17], [18].

Detailed studies have shown that different classes of Topo II poisons differ in their activities, even though they all act through the same basic mechanism of retarding the religation reaction at the end of the catalytic cycle [13], [19]. This has previously been extensively analyzed in a system of *Drosophila melanogaster* tissue-culture cells, in which we initially characterized sites of Topo II/chromatin interactions, both *ex vivo* and *in vitro*, at nucleotide resolution [12], [20], [21]. Different classes of poisons (epipodophyllotoxins, acridines and anthracyclines) typically differ in the cleavage sites they induce, as well as in their stability and reversibility [22], [23]. The strict molecular basis for these differences remains poorly understood. In the case of etoposide and teniposide, which exert strong cytotoxic effects and stimulate Topo II-mediated cleavage in cells, the generation of derivatives with more specific activities and/or a restricted subset of genomic targets constitutes an attractive strategy towards the development of new, more effective drugs.

F14512 is a novel epipodophyllotoxin derivative which possesses antitumor properties in mice harboring human tumors [24], [25]. Using both enzymatic and cellular assays and similar to other previously characterized epipodophyllotoxin-based compounds, F14512 has been shown to inhibit Topo II. F14512 differs from its analogues by the presence, on the epipodophyllotoxin moiety, of a polyamine chain, spermine in this case. Polyamines, such as spermine and spermidine, are natural polycationic molecules required for cell growth [26]. Tumor cells have a higher requirement for polyamines relative to normal quiescent or cycling cells and often overexpress ornithine decarboxylase, the rate-limiting enzyme in polyamine biosynthesis [27], [28], [29], [30]. In addition, to accumulate sufficient polyamine amounts required for their growth, tumor cells also increase their uptake of polyamines from the extracellular compartment *via* the Polyamine Transport System (PTS) [31], [32]. We previously showed that F14512 was preferentially imported by PTS^+^ human tumors xenografted in mice [24]. Moreover, the polyamine targeting moiety of F14512 endows it with significantly higher antitumor properties than the structurally analogous etoposide, in which a sugar is found instead of the spermine chain [24], [25]. In the test tube, in the presence of purified Topo II, F14512 also proved to be markedly more potent than etoposide at inhibiting the enzyme and generating DNA double strand breaks [24]. Surprisingly, this higher potency of F14512 is not reflected by a higher frequency of DNA cleavage induced in treated cells [25]. We attempted to further dissect the possible intricacies of F14512 activity on DNA and chromatin, presumably due to the sole presence of its spermine moiety, and which could explain these apparently contradictory observations. Because of the presence of spermine in the F14512, we also decided to compare this novel compound, in addition to etoposide and teniposide, to the previously described TOP-53, an epipodophyllotoxin derivative that contains a structurally analogous alkyl-amide moiety and possesses anti-tumor activity [33], [34].

Because of the inherent difficulty of addressing such a question in classical cellular models, we report here on the use of a fully integrated *Drosophila melanogaster* model system whose evident complexity is offset by the powerful genetic tools it provides. Using assay systems and loci previously characterized *in vitro* and *in cellulo* for their response to Topo II inhibitors such as teniposide, we tested F14512 for possible differences with its polyamine-less analogs. In addition, we extended our analysis to *in vivo* studies in this organism, where the use of a defined genetic background revealed some unique features of F14512 that provide new insights in the properties of this novel drug. Our results also demonstrate that it is possible to use the *Drosophila melanogaster* model organism as an assay system that provides a low-cost, rapid and effective complement to mammalian models and permits the elucidation of fundamental mechanisms of action of candidate drugs of therapeutic interest in humans.

## Methods

### Drosophila melanogaster fly and cell culture

Flies were grown at 22°C on standard cornmeal-glucose-yeast medium. An Oregon R laboratory stock was used as a wild-type control strain. A laboratory stock of the *white-mottled* inversion (ln(I)*w^m4h^*), referred to as *w^m4h^* here, was maintained at a growth temperature of 18°C. *Drosophila* Schneider S2 and Kc cells were grown at 24°C in Schneider *Drosophila* medium (Invitrogen) and Echalier's D22 medium (Sigma), respectively, supplemented with 5% heat-inactivated newborn calf serum. Under these conditions, S2 and Kc cells have doubling times of approximately 26 and 24 hours, respectively. S2 cells were grown at sub-confluence in 75-cm^2^ flasks or in the 2-cm diameter wells of multi-well plates. Kc cells were grown in spinner cultures at densities ranging between 2×10^6^ to 4×10^6^ cells/ml and transferred to multi-well plates (2-cm diameter wells) for drug treatments. When used, distamycin A_3_ (Sigma) was added to the culture medium for 60 minutes, followed by addition of Topo II poisons at the concentrations indicated in the text. Incubations were continued for an additional 30 minutes (cleavage experiments) or for up to 96 hours (cytotoxicity experiments). The procedure used for cleavage experiments is detailed below.

### Drugs

**Figure 1:**
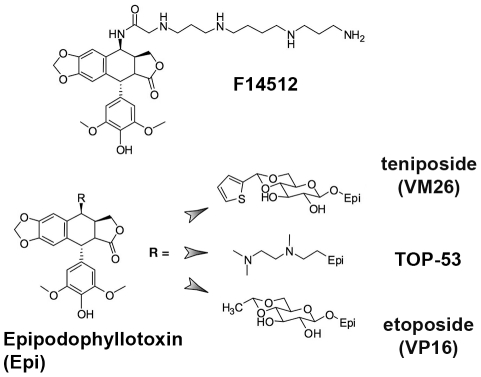
Structure of podophyllotoxin derivatives used in this study. Structure of F14512. Epipodophyllopoxin is shown to the left, with the substitution of different R groups leading to teniposide (VM26), TOP-53 and etoposide (VP16).

Etoposide, TOP-53 and F14512 were obtained from Pierre Fabre Medicament and teniposide from Bristol-Myers Squibb. The detailed synthesis of F14512, based on previously reported chemical procedures [35], will be described elsewhere. The drug TOP-53 (initially developed by Taiho, Japan) is an epipodophyllotoxin derivative that contains a [[(dimethylamino)ethyl]-N-methylamino]ethyl group reminiscent of the polyamine chain of F14512 (Figure 1) [33], [34]. All compounds were prepared as 5 mM stock solutions in DMSO. Water-soluble F14512 was also prepared in DMSO to equalize solvent concentrations in all experiments, except for drug feeding where it was dissolved in water.

### *In vitro* Topo II cleavage experiments

**Figure 2:**
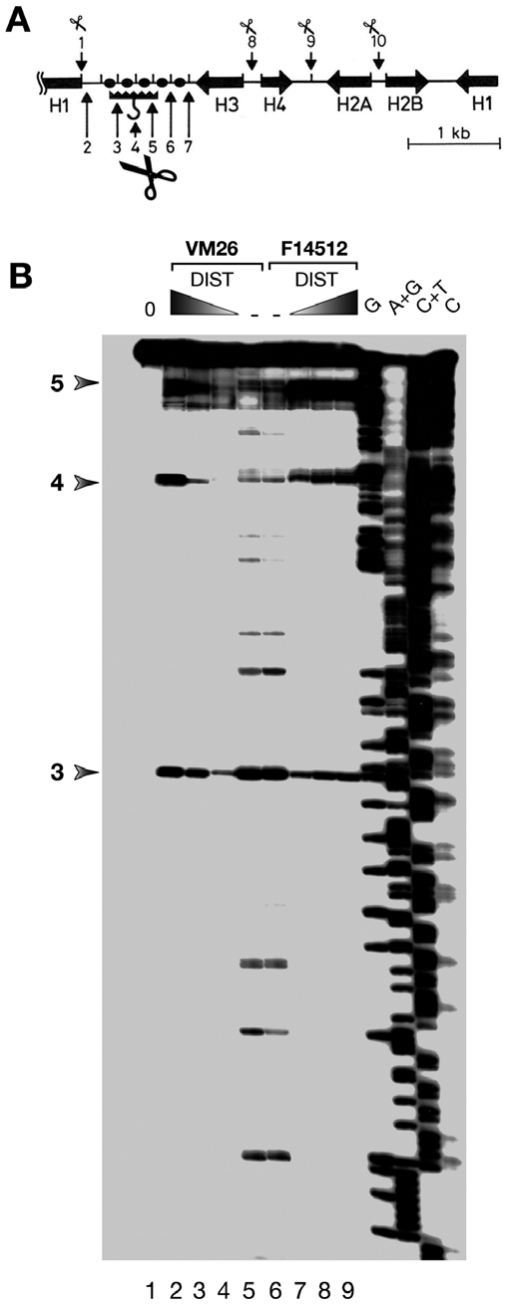
*In vitro* drug-induced cleavage in the *Drosophila* histone-gene repeat. (A) Schematic representation of the 5-kbp major histone-gene locus, which contains the 5 genes encoding core and linker histones. The bar with a hook represents the scaffold-associated region (SAR) located in the intergenic spacer between the H1 and H3 genes [74]. It is cleaved by Topo II (denoted by scissors) in the linker regions of 5 positioned nucleosomes (black circles). Other sites (1, 2 and 8–10) map to DNAse I-hypersensitive sites at the 5′ and 3′ ends of the histone genes (see [21] for details). Sites 3-5 mapped at nucleotide resolution by direct genomic sequencing [21], [22]. (B) *In vitro* cleavage induced by teniposide (VM26) and F14512. A uniquely end-labeled DNA clone spanning the histone-gene repeat was incubated with Topo II as described in Materials and Methods in the presence of no drug (lane 1), 50 µM teniposide with 0, 5, 10 or 25 µM distamycin (lanes 5, 4, 3 and 2, respectively) or 5 µM F14512 with 0, 5, 10 or 25 µM distamycin (lanes 6, 7, 8 and 9, respectively). The 4 lanes at the right of the gel are Maxam-Gilbert sequencing reactions of the labeled fragment. Cleavage is induced at numerous identical sites by teniposide and F14512 in the absence of distamycin (compare lanes 5 and 6). Increasing concentrations of distamycin redirect cleavage induced by both drugs to sites 3–5 marked by arrows to the left of the gel and which correspond exactly to the sites cleaved *in cellulo*.

*In vitro* cleavage experiments (Figure 2) were performed exactly as described [20], using 2 ng (10^4^ cpm) of a cloned uniquely end-labeled *Drosophila* 5-kb histone-repeat DNA fragment and 200 ng of sonicated salmon sperm DNA (average size ∼2 kb) as competitor. These conditions optimize the specificity of the cleavage reaction. 50-µl reaction mixtures were incubated for 15 minutes with 0, 5, 10 or 25 µM distamycin as shown in Figure 2B, before addition of 0.5 µl of human Topo IIα purified using an expression vector kindly provided by Dr. J. L. Nitiss. Incubation was continued for 5 minutes at 37°C to allow binding to occur before addition of teniposide (50 µM) or F14512 (5 µM) and ATP (1 mM). A stop mix was added (1% SDS, 5 mM EDTA, 100 µg/ml proteinase K in a 300 µl final volume brought up with TE) after a 5-minute additional incubation. Following incubation for 3 hours at 55°C, DNA samples were purified by organic extractions and ethanol purification in the presence of 0.2 M NaCl and electrophoresed on 5% acrylamide/6M urea sequencing gels in TBE at 2000 volts. Gels were fixed in 10% acetic acid, dried and exposed to Kodak BioMax MS-1 film with BioMax MS intensifying screens. Maxam-Gilbert chemical sequencing reactions performed on the same labeled histone-repeat fragment were loaded on the gels. Because the 5′ end of the fragment (as shown on the map in Figure 2A) was labeled, sequencing gels and sequencing reactions allow the precise assignment of cleavage sites 3–5. Similar experiments not shown here were performed with a cloned uniquely end-labeled SAT III monomer fragment (see below), yielding comparable results.

### *Ex vivo* SAT III cleavage experiments

**Figure 3:**
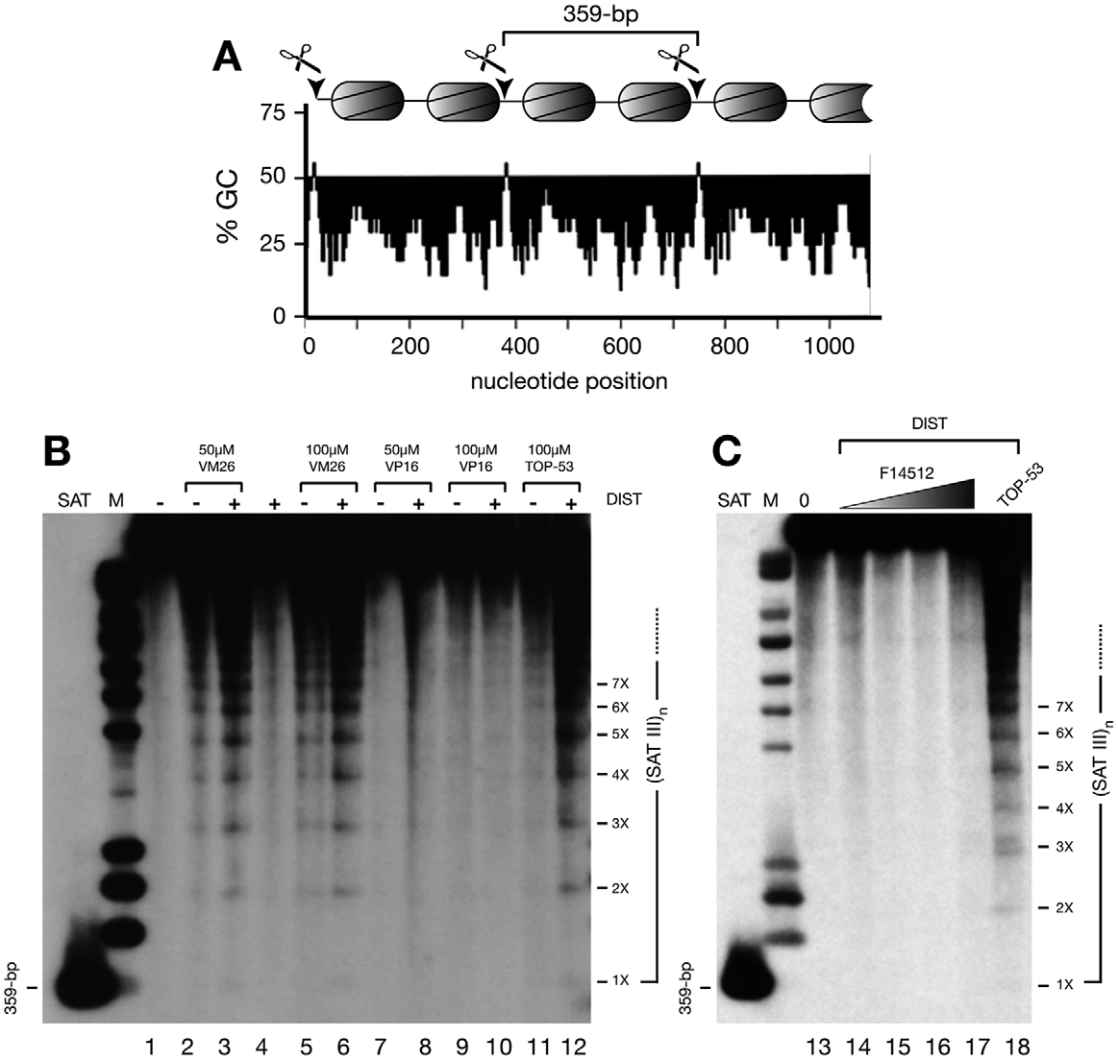
Cellular profiles of drug-induced Topo II-mediated cleavage in SAT III repeats of the *Drosophila* X chromosome. (A) Structure of SAT III repeats. 359-bp SAT III repeats accommodate 2 nucleosomes per repeat. One of the two nucleosomal linkers located in each repeat contains a short GC-rich sequence that serves as a highly specific target for Topo II cleavage *in vivo*, as denoted by scissors. (B) Schneider S2 cells were treated for 30 minutes with teniposide (VM26), etoposide (VP16) or TOP-53 at the concentrations shown, in the absence (-) or presence (+) of distamycin (DIST, 25 µM). Purified DNA was electrophoresed without prior restriction enzyme digestion, transferred to a nylon membrane and hybridized to a labeled SAT III probe. 359-bp: the SAT III monomer (SAT) used as a probe; M: molecular weight standards. (**C**) S2 cells were treated for 30 minutes with no drug (0, lane 13), F14512 (5, 10, 25 or 50 µM, lanes 14–17) or 50 µM TOP-53 (lane 18), in the presence of 25 µM distamycin (DIST). Omission of the latter gave identical results (not shown). DNA samples were processed as described above. SAT: the 359-bp cloned fragment used as a probe; M: molecular weight standards.

Approximately 5×10^6^ exponentially growing S2 and Kc cells, cultured as described above, were incubated with Topo II poisons for 30 minutes. In some experiments, cells were first treated with distamycin (25 µM) before addition of teniposide, etoposide, TOP-53 or F14512 added at the concentrations shown in the Figure 3. After a 5-minute centrifugation (3000 x *g*), cell pellets were resuspended in PBS supplemented with drugs, MgCl_2_ (5 mM) and ATP (1 mM) before sequential addition of SDS (1% final concentration), EDTA (5 mM final concentration) and proteinase K (Roche) to 200 µg/ml. Samples were gently mixed following addition of each component to the lysis mix, which was incubated for 3 hours to overnight at 55°C. DNA samples were purified by organic extractions and ethanol precipitation. Washed and dried DNA pellets were resolubilized in 20 µl TE. 10 µl aliquots were ran on 1.2% agarose gels in TBE (35 volts for 14 hours). After staining with ethidium bromide and photography, gels were transferred to charged nylon membranes and hybridized to a radioactively labeled cloned SAT III 359-bp monomer exactly as described [37], except that GeneScreen Plus membranes (Perkin-Elmer) were used instead of Hybond-N+ (GE Healthcare). Filters were washed at high stringency (0.1x SSPE, 1% SDS at 65°C) and exposed to Kodak BioMax MS-1 film with BioMax MS intensifying screens. Exposure times ranged between 0.5 and 14 hours. The procedure used for the global detection of Topo II-induced cleavage in S2 cells is detailed below.

### Global shotgun search for specific F14512-induced chromatin cleavage

**Figure 4:**
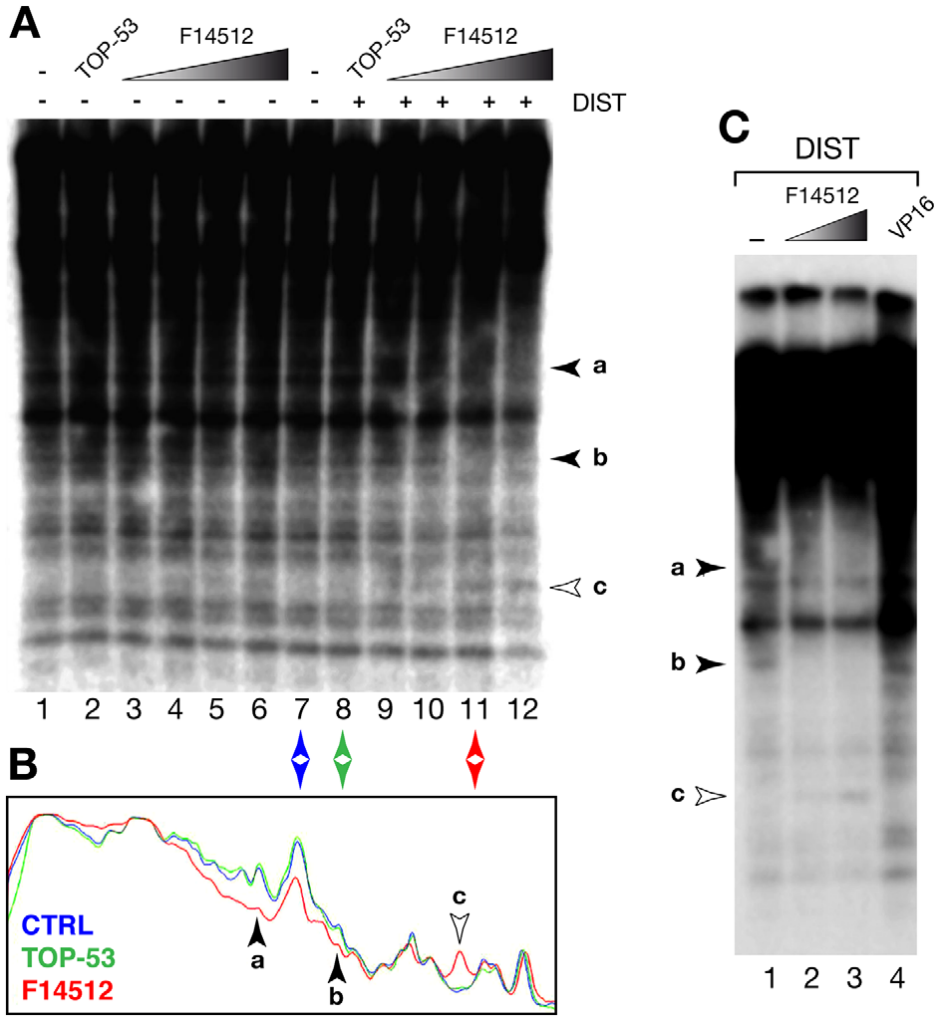
Evidence for F14512-specific Topo II-mediated cleavage in moderately repeated *Drosophila* sequences. (A) *Hin*dIII-digested DNA samples from S2 cells treated for 30 minutes with no drug (lanes 1and 7), F14512 (5, 10, 25 or 50 µM, lanes 2–6 and 9–12) or 50 µM TOP-53 (lanes 2 and 8), in the absence (lanes 1–7) or presence (lanes 8–12) of 25 µM distamycin (DIST). Purified DNA samples were hybridized to a labeled *Hin*fI-digested whole genomic DNA probe that had been pre-hybridized as described in Materials. The a–b–c fragments indicated to the right of the gel are described in the text. (B) Densitometric scan of the autoradiograph shown in (A) for lanes 7, 8 and 11. Regions corresponding to fragments a, b and c are indicated by arrowheads. (C) S2 cells were treated for 30 minutes with no drug (-), F14512 (25 or 50 µM) or 50 µM etoposide in the presence of 25 µM distamycin (DIST). Purified DNA samples were processed as described in panel A. Fragments a, b and c are indicated by arrowheads to the left of the gel, which provides better resolution of the a–b regions.

DNA from S2 cells treated with Topo II poisons in the absence or presence of distamycin was prepared as described above. Purified DNA samples (1 µg) were digested with *Hin*dIII and electrophoresed on 1.2% (Figure 4A) or 0.8%. agarose/TBE gels (Figure 4C). After transfer to GeneScreen Plus membranes, pre-hybridization was performed exactly as described [37] for 2 hours at 42°C in the presence of sonicated and denatured Kc genomic DNA (50 µg/ml) together with sonicated, denatured salmon sperm DNA carrier. During the pre-hybridization step, *Hin*fI-digested Kc genomic DNA (200 ng) was radiolabeled using a random-priming kit (Perkin Elmer) and α-^32^P-dATP (3000 Ci/mmole, GE Healthcare). The labeled whole genomic probe was denatured by boiling for 10 minutes and incubated with 10 µg of cold sonicated (average size ∼1 kb) S2 genomic DNA in 800 µl of 0.12 M sodium phosphate, 1 mM EDTA. Under these conditions, determined empirically using known C_o_t curves of *Drosophila* genomic DNA, incubation at 68°C for 35 minutes results in the rehybridization of snap-back DNA (simple repeats widely spread in the *Drosophila* genome) as well as of C_o_t1 DNA (very high-abundance tandemly repeated sequences). Control experiments have shown that these conditions suppress >80% of the normally observed SAT III cleavage ladder. The pre-hybridized probe was added to the nylon membrane and hybridization of highly (dispersed) to moderately repeated (tandem or dispersed) sequences (C_o_t2 DNA) allowed to proceed overnight before stringent washing and exposure to film (12 to 24 hours) as described above. The resulting signal that can be observed (Figures 4A and 4C) corresponds to the genome-wide pattern of moderately repeated *Hin*dIII fragments or of dispersed (non-tandem) highly repeated *Hin*dIII fragments. The slow kinetics of DNA hybridization onto a solid medium like the nylon membrane (rather than in solution) effectively largely suppress the visualization of single-copy sequences, which otherwise would produce a smear obscuring the distinct pattern of repeated *Hin*dIII fragments.

### Drug treatments and analysis of cytotoxic effects

**Figure 5:**
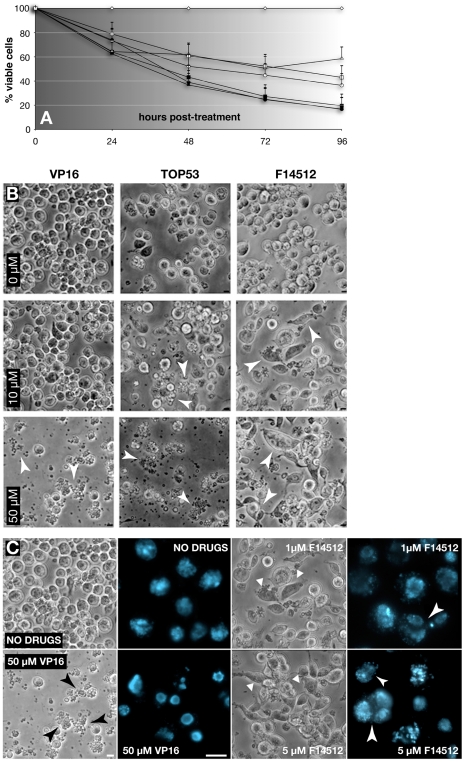
Growth inhibition of *Drosophila* Kc cells by F14512 and characteristic cytotoxic effects. (A) Antiproliferative properties of F14512 and reference Topo II poisons, measured through viable cell counting (Vi-CELL) in exponentially growing *Drosophila* Kc cells. Drugs were used at the following concentrations: F14512 1 µM (-□-) and 50 µM (-▪-); etoposide (VP16) 1 µM (-Δ-) and 50 µM (-▴-); and TOP-53 1 µM (-○-) and 50 µM (-•-); control cells (-◊-) were treated with 0.1% DMSO alone. Viability was measured after the treatment times shown. Mean values +SD of three independent experiments are reported. (B) Phase-contrast micrographs of Kc cells treated as shown with etoposide (VP16), TOP-53 and F14512 and photographed after 24 hours. Arrowheads point to aggregates of apoptotic debris (etoposide VP16, TOP-53) or to elongated and/or multinucleated cells (F14512). Bar: 5 µm. Note that F14512 treatment yields no evident sign of apoptosis at the concentrations used here. (C) Phase-contrast or fluorescence micrographs of intact Kc cells and DAPI-stained fixed cells, respectively, after 24 hours of treatment with etoposide (VP16) or F14512 at the concentrations shown. The black arrowhead in the etoposide-treated photograph indicates apoptotic bodies, DAPI-stained samples show signs of hypercondensed chromatin. Low concentrations of F14512 (1 or 5 µM) again yield large adherent, elongated and multinucleated cells, DAPI staining shows evidence of internuclear chromatin bridges (white arrowheads) Bar: 5 µm.

Cell viability (Figure 5A) was measured in response to exposure to F14512 and reference Topo II inhibitors using the method of viable dye exclusion by living cells. Briefly, 2.5×10^6^ Kc cells were seeded in a 12-well plate 24 hours before addition of F14512 or the reference Topo II inhibitors. The number of viable cells was then determined 24, 48, 72, and 96 hours post-treatment using the Vi-CELL analyser (Beckman Coulter). The percentage of viable cells was then calculated with reference to the control culture exposed to the drug vehicle 0.1% DMSO, and normalized to 100% for each time point explored.

For observation of drug-mediated cytotoxic effects (Figures 5B and 5C), Kc cultures were treated with teniposide, etoposide, TOP-53 or F14512 at the concentrations indicated in the text for up to 72 hours. Live cultures were monitored by phase-contrast microscopy starting at 2 hours and photographs taken using the 40x objective of a Nikon Eclipse TS100-F microscope equipped with a Spot Insight Color CCD camera. For examination of nuclei, aliquots of cells were withdrawn and fixed in PBS containing 0.1% Triton X-100 and 4% paraformaldehyde for 10 minutes at room temperature. Fixed cells were transferred to a microscope slide, the excess fixative carefully removed with a Kimwipe and the nuclei stained with DAPI (10 µg/ml) in anti-fade solution [36]. Coverslips were sealed onto the slides with nail polish and the samples photographed under UV illumination using a Leica DMRB microscope equipped with a CoolSnap HQ CCD camera. Photographs were processed with Adobe Photoshop to render grey scale images in false colors and corrected for black balance.

### *In vivo* drug-feeding experiments (“oral administration”)

**Figure 6:**
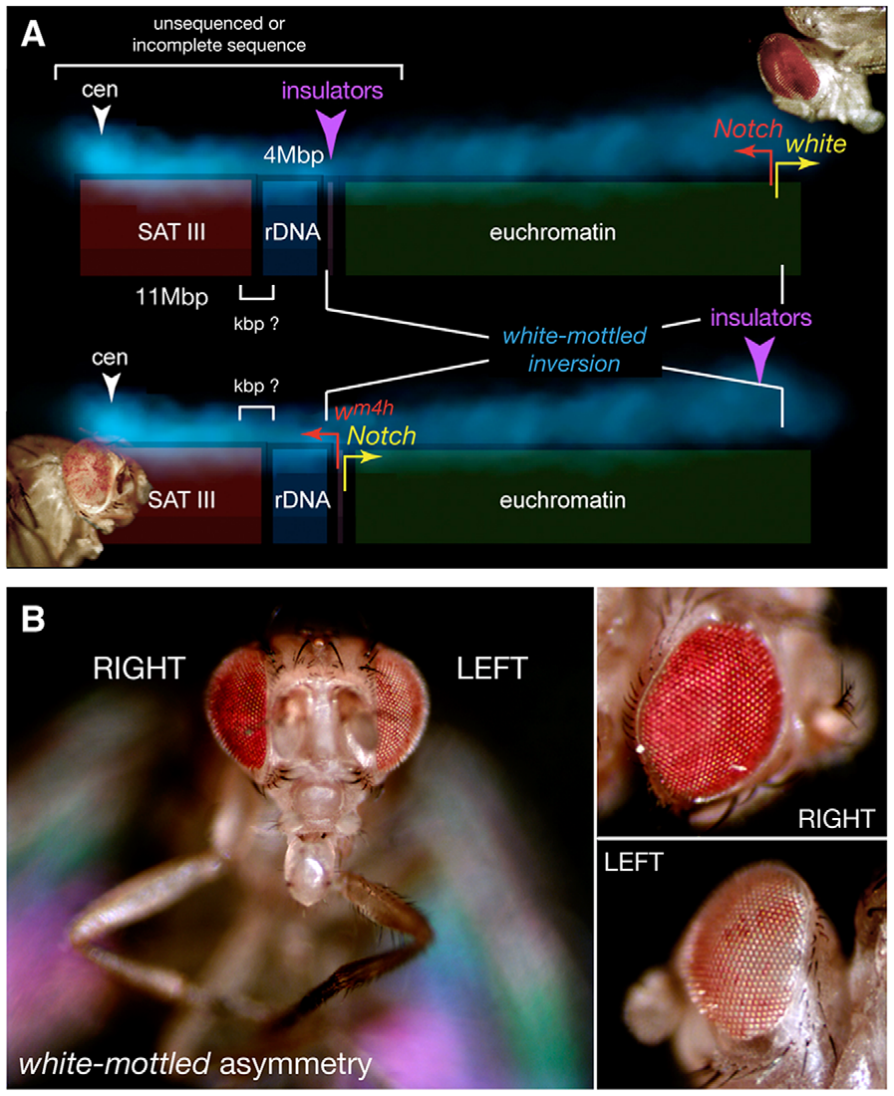
The *Drosophila white-mottled* inversion: a classical system to study long-range regulation of gene expression. (A) Schematic representation of the *Drosophila* X chromosome and of the *white-mottled* (*w^m4h^*) inversion that juxtaposes the *white* gene to a heterochromatin context, in this case within the 4-Mbp rDNA locus adjacent to the SAT III array. Different features of the proximal end of the chromosome, most of which remains unsequenced, are discussed in the text. Stochastic silencing of *white* leads to a mottled eye color (bottom left corner) compared to the wild-type red adult eye (top right corner). This phenomenon corresponds to the classical definition of position-effect variegation (PEV). The nearby *Notch* gene is similarly affected under certain growth conditions (see text for details). (B) Intrinsic loss of symmetry resulting from *white* and *Notch* PEV in *white-mottled* flies. The fly shown is a representative example of an adult phenotype observed at 1–2% frequency in the *w^m4h^* line when grown at 18°C. The facing view illustrates a characteristic mottled color of the left eye, but a wild-type red color on the right. This is more clearly seen in the right and left close-ups shown to the right. This intrinsic propensity for asymmetry in *w^m4h^* PEV manifestations most likely results from *Notch* misregulation and is important to understand the one-sided phenomena illustrated in Figure 8.

**Figure 7:**
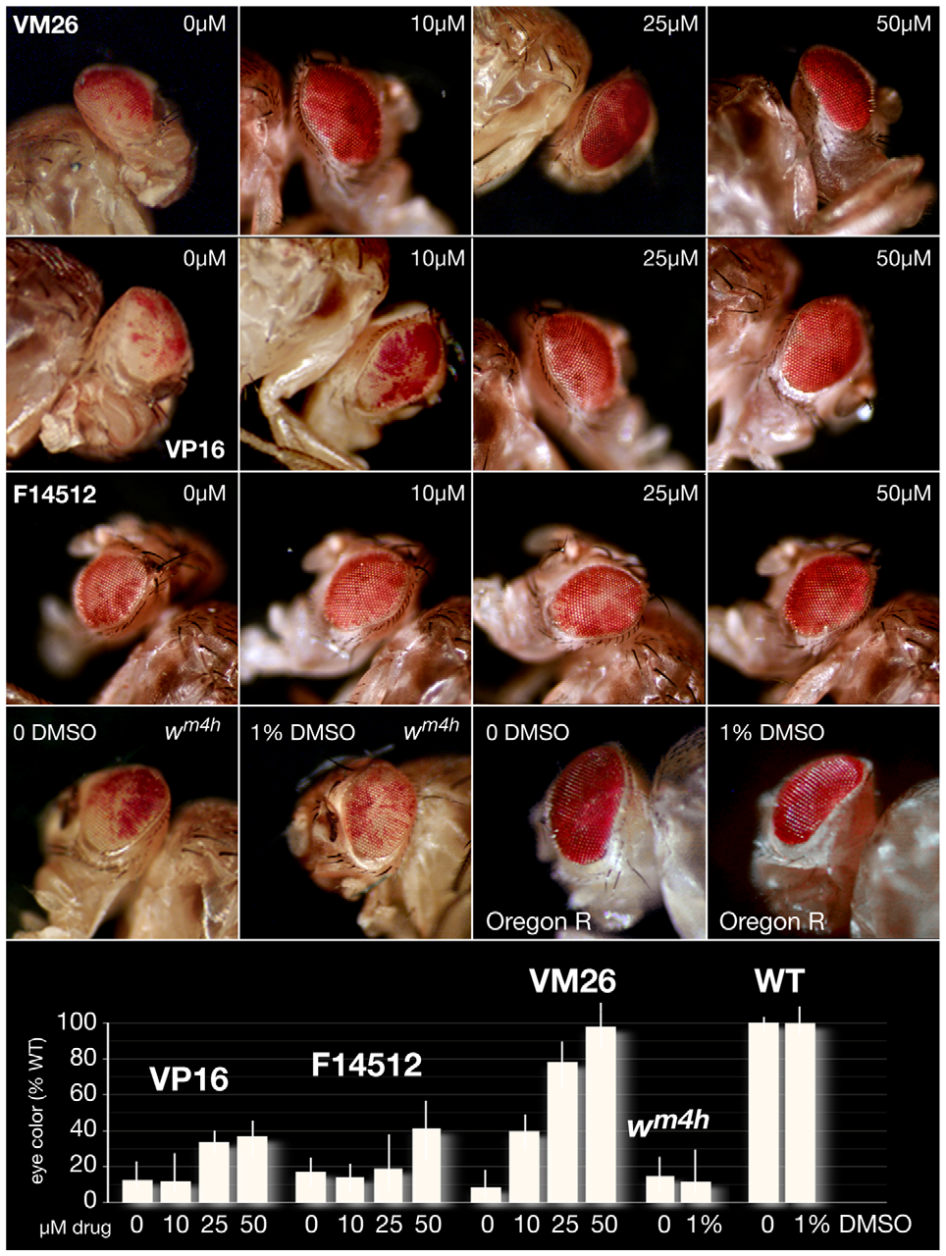
Suppression of *white-mottled* PEV by Topo II poisons. Photographs show representative eyes of 5-day old *w^m4h^* females hatched from late L3 larvae fed etoposide (VP16), F14512 or teniposide (VM26) at the indicated concentrations. A comparison of the no-drug controls shown in the left column highlights the intrinsic heterogeneity of the *white-mottled* phenotype. Etoposide and F14512 partly suppress *white* PEV at higher concentrations and display an apparent threshold effect at 25 µM or 50 µM, respectively. Teniposide feeding leads to full dose-dependent suppression of PEV. Controls (*w^m4h^* or wild-type Oregon R larvae fed 0 or 1% DMSO used to dilute the drugs, a concentration that corresponds to that used in 100-µM drug treatments) are shown in the bottom row. The graph at the bottom shows the corresponding quantifications of extracted eye pigment and represents mean values obtained from three independent experiments performed in duplicate (30 5-day old females scored for each condition). Results are represented as % pigment relative to Oregon R wild-type (WT) flies fed 1% DMSO. Error bars represent the experimental error. Note that the toxicity of F14512 feeding at 25 and 50 µM only permitted analysis of 10 females for each experiment, most likely accounting for the substantial variations observed (p<0.2 in contrast to p<0.01 for other drug treatments).

**Figure 8:**
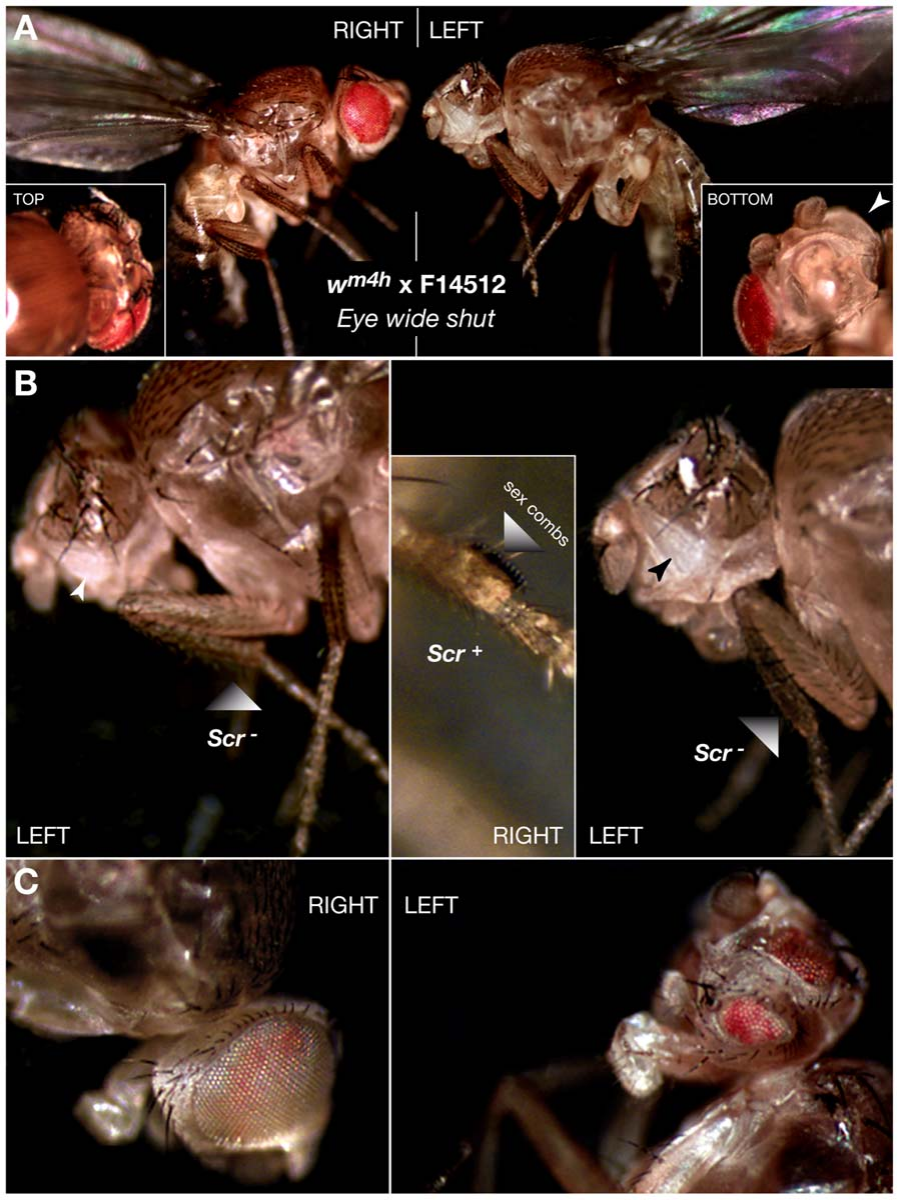
*Eye wide shut*: specific loss- and gain-of-function phenotypes after F14512 oral administration to *white-mottled* larvae. (A) Different views of a representative *Eye wide shut* adult male recovered from feeding 25 µM F14512 to *w^m4h^* larvae. The right side shows suppression of PEV by the drug while, on the left side, the eye has been replaced by a first thoracic treatment, best explained by a left side-only *Antp* gain-of-function and/or *ey* loss-of-function. The latter is also suggested by the presence of a small remnant of an eye (arrowhead in bottom view; see also panel C). The striking asymmetry of the F14512-induced *Eye wide shut* phenotype is consistent with the propensity for loss of right/left symmetry in the *w^m4h^* line (Figure 6B). (B) Close-up views of the left side of F14512-induced *Eye wide shut* male adults. Homeotic transformation of the left eye into a first thoracic segment can be clearly seen, white arrowheads indicate a small remnant of an eye. The inset shows an enlarged view of a normal right side, with male-specific sex combs on the first tarsal segment of the fore leg indicated by the shaded arrowhead. Sex combs are absent on the left side where the eye is also transformed in a first thoracic segment, suggesting a loss-of-function of the homeotic gene *Sex combs reduced* (*Scr*). Note that *Scr* is closely linked to *Antp* and is also subject to Polycomb-mediated regulation via mechanisms that implicate Topo II. (C) Asymmetric *ey* loss-of-function in an F14512-fed *w^m4h^* adult male. Feeding lower concentrations (10 µM) of F14512 to *w^m4h^* larvae occasionally leads to the recovery of other phenotypes at a frequency of about 5%. The right side of the fly again shows a normal eye with a characteristic mottled color. The left eye has been diminished to two distinct smaller eye structures characteristic of partial *ey* loss-of-function.

The chief characteristics of the *white-mottled* (*w^m4h^*) line used in these experiments are illustrated in Figure 6. Batches of 30 late third-instar L3 *w^m4h^* or wild-type Oregon R larvae were transferred to agar plates containing 0, 10, 25, 50 or 100 µM etoposide or teniposide or with 1, 5, 25, 50 or 100 µM F14512 and yeast paste supplemented with the same concentration of the drugs. Late L3 larvae feed actively and can be easily picked from a *Drosophila* culture as they are the most mobile. This insures that the larvae will fairly uniformly eat the drug-laced yeast paste, the drug present in the agar preventing dilution. Treatment with 100 µM teniposide or 50 µM F14512 led to >90% lethality. No lethality (<10%) was observed following treatment with lower concentrations of the drugs (up to 75 µM teniposide and 25 µM F14512), which led, however to developmental delays during pupation. Eye pigment levels (Figure 7) were measured from the heads of 30 5-day old adult female flies after photography as previously described [36] or, in the case of F14512-fed flies, from 10 female heads as drug concentrations of 25 or 50 µM lead to approximately 50-70% lethality. Males were examined as early as 1 or 2 days following hatching, as *Eye wide shut* adults die within 2 days. This provides enough time to score for the gain- or loss-of-function phenotypes shown in Figure 8. Flies were photographed using a Leica MZ-FLIII binocular microscope equipped with a CoolSnap CCD camera.

## Results

### F14512 is a potent inducer of Topo II cleavable complexes on naked DNA

F14512 is a novel epipodophyllotoxin-based Topo II inhibitor vectorized towards cancer cells through its spermine moiety (Figure 1). The relationship between its potent cytotoxic effects [24] and its activities as an inducer of Topo II-mediated cleavage, which differ *in vitro* and *in cellulo*, remains unclear. F14512 was previously shown to induce DNA cleavage when incubated with purified human Topo II and a cMYC DNA probe, and was shown to be more potent than teniposide [24]. Using the *Drosophila melanogaster* model system to further characterize the specific features of F14512, we first performed similar experiments using a cloned DNA fragment corresponding to the histone-gene repeat of *Drosophila* (Figure 2A). Teniposide-induced cleavage sites have been previously characterized in this 5-kbp sequence at nucleotide resolution *ex vivo* [21], [22] and *in vitro*. DNA incubation with human Topo IIα before addition of teniposide or F14512 yielded essentially identical cleavage patterns (Figure 2B, compare lanes 5 and 6). Note, however, that F14512 was used at a 10-fold lower concentration (5 µM) than teniposide (50 µM) to obtain comparable extents of cleavage of the 5-kbp fragment. Hence, addition of a spermine moiety to a podophyllotoxin-based molecule does not, in this system, alter the basic properties of the Topo II poison and F14512 retains an identical selectivity for cleavage-site selection. It is however likely, given the 10-fold differences in drug concentrations used to yield comparable extents of cleavage products that the presence of spermine promotes the formation of a more stable cleavable complex. Such a possibility is supported by the previously characterized interaction of F14512 with DNA [24].

However, the presence of the polyamine moiety did not perturb the previously characterized sensitivity of Topo II-mediated cleavage to distamycin [38], a DNA minor-groove binder specific for stretches of 4 or more dA•dT base pairs [37]. As shown in lanes 2–4 and 7–9, addition of increasing concentrations led, after treatment with either teniposide or F14512, to a striking restriction of Topo II cleavage down to three major sites (marked 3–5 to the left of the gel) that correspond, to the nucleotide, to those cleaved after *ex vivo* treatment of Kc cells with teniposide, and where distamycin potentiates Topo II-mediated drug-induced cleavage [37]. Hence, the spermine moiety does not perturb drug/Topo II interactions in the presence of distamycin, which can redirect the sequence-specific localization of the protein [3]. In accordance with the observation that these identical patterns require 10-fold less F14512 than teniposide, we conclude from these results that the polyamine moiety might serve to stabilize ternary drug/DNA/protein complexes, a hypothesis next tested using a well characterized *ex vivo* system, where polyamine interactions with chromatin might affect sites of drug-induced Topo II cleavage.

### Differential *in cellulo* induction of Topo II-mediated cleavage in the highly repeated SAT III region of the X chromosome

*In cellulo*, our experimental system is based on the AT-rich 1.688 g/cm^3^ satellite III (SAT III) repeat, a 359-bp sequence repeated approximately 30,000 times in tandem near the proximal tip of the *Drosophila melanogaster* X chromosome (see Figure 6A below). SAT III repeats are highly enriched for Topo II [39] and constitute a hotspot for DNA cleavable-complex formation induced by teniposide, but not by amsacrine or anthracyclines [21], [23]. These observations are consistent with the selective association of Topo II with A+T-rich sequences *in vitro* [20] and are accompanied by a similarly preferential interaction with these sequences *ex vivo*, as shown for teniposide-stimulated cleavage of SAT III repeats [37]. This huge 11-Mbp target, whose structure is schematized in Figure 3A for 6 adjacent 359-bp monomers can be used as a convenient model system to analyze Topo II cleavage *in* or *ex vivo*. The high abundance of the corresponding sequence (almost 5% of the whole genome in diploid tissues) facilitates analysis of rare Topo II cleavage products. As each 359-bp repeat contains a unique Topo II cleavage site located in one of two nucleosomal spacers (Figure 3A), partial cleavage results in a ladder of products of (359-bp)_n_ size.

As shown in Figure 3B, a ladder of SAT III cleavage products readily appeared following a short 30-minute treatment of cultured Kc cells with Topo II poisons such as teniposide or TOP-53, but was largely absent in DNA isolated from cells treated with etoposide. In all cases, cells were treated for 30 minutes with each drug at the indicated concentrations, in the absence or presence of distamycin, which renders SAT III sequences more accessible to Topo II. This treatment led to an expected increase [37] in the intensity of the 359-bp cleavage ladder (compare lanes 3, 6 and 12 to lanes 2, 5 and 11). In contrast and similar to the lack of activity of etoposide, we could not detect F14512-induced Topo II cleavage in the SAT III locus, irrespective of the presence of distamycin (Figure 3C, lanes 13-17). This outcome implies that the cellular Topo II-directed activity — if any — of F14512 is comparable to that of etoposide (see Figure 3B), but differs from that of its closest parent, TOP-53. However, we cannot exclude the possibility that either drug is active at this locus, but yields extremely short-lived cleavable complexes whose instability makes it impossible to observe detectable cleavage products. Alternatively, the presence of the spermine moiety in F14512 might direct it to a discrete subset of chromosomal sites, which in this case might differ from those induced by etoposide, teniposide or TOP-53.

### Shotgun search for F14512-stimulated Topo II in *Drosophila* cells

Looking for evidence of F14512-induced Topo II-mediated cleavage in a >200-Mbp genome is a daunting task. A low frequency of cleavage sites located on average every 200 kbp (close to 10-fold less than estimates in vertebrates, in line with the overall rarer cleavage induced by F14512 in mammalian cells) still corresponds to a range of 1000 potential targets. We chose instead to use a genome-wide approach similar to that used almost 20 years ago for the discovery of Topo II cleavage of SAT III sequences [21]. In this case, we had used a radio-labeled probe prepared from total *Drosophila* genomic DNA and hybridized it to undigested genomic DNA extracted from teniposide-treated cells. The kinetics of hybridization of repeated sequences permitted in this case the detection of cleavage products that were eventually shown to originate from SAT III repeats. However, those, as shown above, are not cleaved following treatment with F14512.

To detect potential F14512-induced cleavage sites in the *Drosophila* genome, we introduced modifications to our original strategy. As hybridization to a whole genomic probe did not reveal any F14512-induced cleavage products, as in the case of SAT III repeats, we chose to digest genomic DNA isolated from untreated or treated cells with a restriction enzyme (*Hin*dIII in this case) before electrophoresis and hybridization to the whole genomic probe. Another modification was a pre-hybridization step for the denatured whole-genome probe which, under controlled conditions, serves to mask hybridization to highly repeated (snap-back simple repeats and C_o_t1) sequences that might otherwise obscure any specific signal (see Methods for details).

Schneider S2 tissue culture cells were used in this case instead of Kc cells as their polyploidy increases copy number per cell and their SAT III repeats are cleaved similarly to those of Kc cells following treatment with teniposide (data not shown). After treatment with TOP-53 or F14512 in the absence or presence of distamycin (see below), genomic DNA was purified, digested with *Hin*dIII and hybridized as described in Methods to the pre-hybridized genomic probe. Results of such an experiment are shown in Figure 4. Note that the numerous bands detected in controls (lanes 1 and 7 of Figure 4A) correspond to moderately repeated genomic *Hin*dIII fragments and not to Topo II cleavage products. Unique sequences only give rise to a low-level random background smear. This approach yielded encouraging results as it readily identified bands whose detection was sensitive to F14512 treatment, demonstrating the usefulness of such a method when seeking putative —and unknown— drug-induced Topo II cleavage sites *in cellulo*. Compared to no-drug (lanes 1 and 7) or TOP-53 (lanes 2 and 8) controls, F14512 treatment resulted in the disappearance of two repeated *Hin*dIII fragments (labeled “a” and “b”) and to the appearance of a new fragment (labeled “c”). We surmise that this new fragment might arise from cleavage of the “a” or “b” fragments, or both. A densitometric analysis of lanes 7, 8 and 11 is shown in Figure 4B. While fragment “a” falls in an overexposed and poorly resolved portion of the gel, a significant decrease in the radioactive signal can be observed in this region, while the disappearance of fragment “b” and the appearance of fragment “c” can be clearly discerned. In addition, this pattern was clearly dependent on the concentration of F14512 used.

A separate experiment is shown in Figure 4C, using a lower-percentage agarose gel and DNA from cells treated with F14512 and etoposide in the presence of distamycin. Compared to no-drug (lane 1) and etoposide samples (lane 4), F14512-treated cells yielded an identical a-b-c pattern, establishing the reproducibility and drug specificity of these results, for which the region spanning fragment “a” is better resolved. Quite interestingly —and unusually for treatment of intact cells with Topo II poisons— this behavior was quite quantitative, possibly indicating a highly specific and potent cleavage activity. Because we know of no other sequence-specific nuclease activities that can be induced in *Drosophila* cells under the conditions used, these results necessarily seem to implicate Topo II in this phenomenon.

Simple interpretations of these preliminary results lead us to propose several important conclusions. First, treatment of *Drosophila* cells with F14512 results in a pattern of DNA cleavage at discrete moderately repeated sites that is not seen following treatment with TOP-53, its closest relative, or with teniposide or etoposide. As teniposide and TOP-53 induce cleavage in SAT III repeats while etoposide and F14512 do not, the a-b-c pattern clearly reflects a property unique to F14512. Importantly, this F14512-specific behavior was amplified in the presence of distamycin (compare lanes 3–6 to 9–12, in Figure 4A, where the disappearance of fragment “b” occurs faster than in the absence of this AT-specific minor-groove binder). Indeed, note that bands a-c are difficult to discern in the absence of distamycin, which opens AT-rich chromatin and makes it more accessible to non-histone chromatin-associated proteins such as Topo II [37]. Another plausible conclusion then is that F14512-induced cleavage in the moderately repeated sequences detected here occurs in generally AT-rich regions, suggesting that the presence of a spermine moiety influences cleavage-site localization in chromatin but not sequence preference [40]. Because Topo II can play a role as a transcriptional regulator [41], [42], [43], [44], F14512-specific targets might give rise to unique changes in patterns of gene expression, which we investigated next by exploring the possible phenotypic effects of this new anticancer drug *in vivo*.

### F14512 inhibits the proliferation of *Drosophila* cells and induces unique morphological changes

Altogether, the presented data demonstrate that F14512 is a Topo II poison that induces an atypical cellular cleavage pattern compared to the reference compounds etoposide, teniposide and TOP-53. We then asked whether this originality translates into different antiproliferative properties of F14512 in *Drosophila* Kc tissue-culture cells, compared to those of related epipodophyllotoxins lacking the polyamine moiety. Viability measurement clearly indicated that all the molecules tested, F14512 as well as the Topo II inhibitors etoposide, teniposide and TOP-53, inhibited proliferation as early as 24 hours after drug addition (Figure 5A). The number of viable cells was further decreased after 48 and 72 hours. In addition, dose- and time-dependent effects were evident for each compound, suggesting that all four drugs exert comparable effects in Kc cells.

F14512 has demonstrated IC_50_ values for proliferation in the nanomolar range for a 72-hour treatment of a series of mammalian cancer cell lines [24]. Kc cells seem then to appear more resistant to F14512. Since these cells are non cancereous one possibility is that they express less PTS, which would expose them to lower intracellular concentrations of compound. However, given the differences highlighted by the cellular cleavage patterns, we asked whether F14512 might exert effects different from those of its related parent drugs.

As a straightforward way of addressing this question, Kc cells exposed to the same compounds were examined by phase contrast microscopy 24 hours post-treatment. As shown in Figure 5B, untreated Kc cells are round and refringent and growing cultures are enriched in doublets of these non-adherent cells. Treatment with either TOP-53 or etoposide at 10 or 50 µM concentrations led to the appearance of apoptotic cells and/or debris in a dose-dependent fashion (arrowheads in Figure 5B), a known and expected effect of prolonged exposure to podophyllotoxins-based Topo II poisons [17], [45], [46]. Concentrations >50 µM resulted in 100% killing over 72 hours of treatment (data not shown). In contrast, treatment with 10 and 50 µM F14512 led a loss of refringence, with doublets becoming rarer in a population of dispersed cells. One characteristic manifestation was the rapid generation of a significant number of giant, multi-nucleated cells (arrowheads, in Figure 5B), which appeared within 2 hours of treatment (data not shown and see Discussion). Treated cells also became adherent and elongated, forming filamentous projections and/or aggregates of seemingly fused cells. These effects became more marked with increasing drug concentrations (compare the 10 and 50 µM treatments). It is significant to note that, even at high F14512 concentrations, we could observe no obvious apoptosis products or cellular debris thereof, in contrast to the effects of etoposide and, significantly, the closely related TOP-53 lacking a spermine moiety.

We investigated these effects in more detail, using lower drug concentrations and by comparing phase-contrast images of intact cultures with those of DAPI-stained nuclei of fixed treated cells. As shown in Figure 5C, F14512 treatment led to effects strikingly different from those of etoposide and that could be observed at concentrations as low as 1 µM. Nuclei became enlarged and more heterogeneously stained. Some nuclei were clearly fused and their chromatin intimately linked. In some cases, anaphase bridges could be observed, suggestive of segregation defects. Other nuclei were regrouped in closely associated aggregates that most likely correspond to multinucleated giant cells observed by phase-contrast microscopy. Small hypercondensed structures that correspond to apoptotic bodies prevalent after treatment with etoposide or TOP-53 could only very rarely be observed. These observations clearly suggest that the mode of action of F14512 and its greater cytotoxicity rest on mechanisms different from those of its close relative TOP-53, or of the etoposide podophyllotoxin used in the clinic.

### The *white mottled* inversion and PEV

We extended our studies to developing *Drosophila* larvae, using a well defined genetic system characterized by H. J. Muller over 80 years ago [47]. As schematized in Figure 6A, the *white-mottled* (*w^m4h^*) chromosomal inversion of most of the euchromatic arm of the X chromosome places the *white* gene next to pericentromeric heterochromatin. *white* encodes a pigment transporter required for the normal red eye color of wild-type flies. Partial inactivation of *white* in *w^m4h^* flies results in a salt-and-pepper (“mottled”) white/red eye color, a phenomenon referred to as position-effect variegation (PEV). PEV reflects the stochastic inactivation of genes placed in or near heterochromatin, the resulting silencing or expression pattern, once established, being then stably propagated to daughter cells. This differential effect is classically attributed to varying extents of heterochromatin spreading into euchromatin regions as a result of the loss of insulator or boundary elements that are thought to prevent such propagation in normal chromosomes ([48], [49] and references therein).

While a mottled eye color is the most obvious and easily scored phenotype of adult *w^m4h^* flies, this line also grows more slowly than wild-type and the extent of eye-color variegation strongly depends on growth conditions. Growth delays might reflect a misregulation of rRNA expression as the rDNA locus lies very close to or even within the proximal breakpoint. The nearby *Notch* gene, 338.5 kbp distant from *white*, is also subject to PEV in *w^m4h^* flies, an effect that is not as strong as on *white* expression and is also sensitive to changes in growth conditions [50]. PEV of *Notch* might account for some abnormal growth phenotypes as its gene product is implicated in a broad array of developmental processes (see http://flybase.org/reports/FBgn0004647.html), including establishment of lateral symmetry [51], [52]. This probably accounts for a striking loss of symmetry of white PEV observed in about 1% of adult *w^m4h^* flies grown at 18°C (Figure 6B) and which extends to the unique phenotypes described below.

### F14512, Topoisomerase II and suppression of *white-mottled* PEV

The *white-mottled* system lends itself to the identification of factors that can perturb Topo II activity in a developing whole organism and of their effects on a readily measurable phenotype. We previously showed that Topo II is a structural and functional component of heterochromatin and can modulate the *white-mottled* phenotype [39]. Accordingly, the eye color of *w^m4h^* flies is uniquely sensitive to the chromosomal localization of Topo II [53] and feeding teniposide to developing *w^m4h^* larvae suppresses *white-mottled* PEV, as shown by the recovery of adults with wild-type eye color [39]. We compared this effect to that of F14512 and etoposide. For the experiments shown in Figure 7, *w^m4h^* late-stage third-instar (L3) larvae were grown overnight with Topo II poisons added to the food at the sub-lethal concentrations shown. Adults were photographed 5 days after hatching, before pigment extraction from the heads of 30 or 10 females (see Methods) and quantification.

Feeding teniposide (10–50 µM, see Figure legend for details) to *w^m4h^* late L3 larvae led to a dose-dependent suppression of the PEV phenotype, with a wild-type eye-color obtained at 50 µM teniposide (see graph at the bottom of Figure 7). Drug concentrations above 100 µM resulted in >90% lethality (data not shown). While etoposide feeding led to a similar lethality, it resulted in only moderate suppression of *white-mottled* PEV. F14512 was found to be significantly more toxic (about 30% hatched adults at 50 µM), but behaved similarly to etoposide in moderately suppressing PEV in the 10–50 µM range, albeit with a relatively wide spread between experiments. We attribute these differences between teniposide and etoposide and F14512 to their differential effects on the stimulation of Topo II cleavage, as in the case of SAT III repeats (see Figure 3 and Discussion). DMSO alone (the solvent for all drugs used here) had no effect on *white-mottled* PEV or on wild-type Oregon R flies. However, we did detect strikingly specific properties of F14512 in this system, consistent with its plausible induction of cleavage at a select subset of genomic sites (see Figure 4), and which we describe next.

### *Eye wide shut*: a unique side effect of F14512 “oral administration”

Feeding F14512 at concentrations above 50 µM led to 100% lethality (no adults hatched). At concentrations of 50 µM, approximately 30% of treated larvae hatched into adults, of which about 10%, which all died within 2 days, showed the remarkable effects illustrated in Figure 8. These dying young adults possess one wild-type eye with normal or near-normal eye color. Quite amazingly, their other eye has been replaced by a first thoracic segment, which also retains a shrunken remnant of a white-colored eye (Fig. 8A, white arrowhead in the inset showing a bottom view of a head). This phenotype affected exclusively the left side of the fly and was only scored in males, denoting a sex-dependent loss-of-symmetry which untreated *w^m4h^* flies appear to be prone to at some frequency (Figure 6B).

Closer views are shown in Figure 8B and reveal additional differences. A disappearance of the male-specific sex combs present on the front legs could also be observed and always occurred on the same side as the transformed eye. Because the most impressive manifestation of oral administration of F14512 to developing *w^m4h^* larvae is the asymmetric homeotic transformation of one eye of affected flies, we refer to this phenotype as “*Eye wide shut*”. The *Eye wide shut* phenotype was reproducible: we carried out 5 series of independent experiments starting with 30 *w^m4h^* L3 larvae in duplicates, each yielding 1 to 2 adults showing these striking abnormalities. Feeding etoposide, teniposide or DMSO never produced these changes, while feeding F14512 to wild-type Oregon R larvae led to no effects other than lethality at high concentrations. Intermediate asymmetric effects were also observed at a lower frequency, as shown in Figure 8C, where a dying adult retains a mottled right eye, while its left eye has been duplicated into smaller structures. As discussed below, these striking phenotypes can be correlated to precise genetic gains- or losses-of-function and all point to an implication of site-specific Topo II dysfunction in the effects we detect using the *white-mottled* model system following treatment with F14512, but not etoposide.

## Discussion

F14512 is a novel targeted Topo II inhibitor possessing strong anticancer activity in mouse models of human tumors [24], [25]. The results reported here demonstrate that, in addition to halting *Drosophila* cell growth in culture, F14512 induces unique features including rapid adherence, flattening and multi-nucleation but, at the concentrations used here, without the signs of apoptosis that are the hallmarks of etoposide and TOP-53, its closest relative. Although we cannot exclude more complex explanations, is appears reasonable to propose that the spermine moiety of F14512 might be responsible for these specific effects. In spite of these profound differences, F14512 remains highly cytotoxic and other possible pathways of growth arrest and cell death are currently under investigation. Recently, F14512-induced senescence has been evidenced with different human tumor cell lines (unpublished data). We note that the rapid appearance of multinucleated cells induced by F14512 treatment is reminiscent of the effects of dexrazoxane (ICRF-187), a potent Topo II catalytic inhibitor [54], suggesting the possibility of similar mechanisms of action. However, unlike dexrazoxane [55], [56], F14512 does not induce apoptosis, even after prolonged treatment (Figure 5). Most importantly, similar to etoposide and teniposide, F14512 has been shown to act as a poison that stabilizes the Topo II cleavable complex [57], albeit with a greater potency that can be attributed to the presence of the spermine moiety. These observations rule out the possibility that, similar to dexrazoxane, F14512 acts as a Topo II catalytic inhibitor.

### Podophyllotoxin derivatives differ in their Topo II poison activities

As expected for an epipodophyllotoxin derivative, F14512 exhibits a potent poisoning activity directed against Topo II and efficiently stabilizes Topo II cleavable complexes when used on a purified DNA target [57]. Using the *Drosophila* histone-gene repeat as a well understood model, F14512 behaved identically to teniposide and showed a similar sensitivity to the effects of distamycin (Figure 2), which affects Topo II localization *in vitro* [20], [38] and *in cellulo* [37]. It is important to note that purified human Topo IIα was used in these experiments. Experiments carried out with purified *Drosophila* Topo II, of which only one isoform exists [58], yielded a slightly different cleavage pattern that reverted exactly to that observed in the presence of distamycin (data not shown), a result identical to that obtained with the single Topo II isoform purified from the yeast *S. cerevisiae* or *S. pombe* [21], [37]. Hence, the cleavage activity and specificity of type II topoisomerases isolated from different organisms may differ but revert to a unique pattern upon addition of distamycin, reflecting a competitive redistribution of the enzyme to drug-free sites that correspond to those cleaved *in cellulo* [3]. In this case, one hallmark of F14512 activity —induction of Topo II-mediated cleavage— is comparable in both *Drosophila* and human cells in that drug treatment results in a cleavage frequency that is markedly lower than that induced by etoposide or teniposide [25]. This suggests comparable modes of action in both organisms, in spite of the absence in one and the presence in the other, of multiple isoforms. These observations also raise the question of which Topo II isoform —α, β or both— is the target of F14512 in human cells. Given the distinct roles of human Topo IIα and β, for example the specific function of the latter in transcriptional regulation [59], identification of the isoform(s) and associated processes targeted by F14512 might help explain the unique potency of the drug. Given that the single *Drosophila* Topo II enzyme shares similarities with both α and β human isoforms and that both Topo IIα and Topo IIβ are efficient targets for F14512 *in vitro* [57], our data do not allow us to answer this question at present. This important point might be addressed in the future by examining Topo IIα and Topo IIβ expression and activity levels in cells selected for resistance to F14512.

Surprisingly, the similar effects of F14512 and teniposide on Topo II-mediated cleavage observed *in vitro* did not translate into cleavage of the SAT III region of the X chromosome of growing *Drosophila* cultured cells (Figure 3C) and F14512 behaved more similarly to etoposide in this case (Figure 3B). This negative result is quite unexpected, as SAT III repeats constitute a massive storage site for Topo II *in viv*o [39] and most likely reflects an intrinsic inability of F14512 to form sufficiently stable ternary complexes with Topo II and DNA at this locus, in accordance with the demonstrated differential activity of different classes of drugs at specific *Drosophila* loci [22], [23]. While we cannot exclude the possibility that this essential enzyme is not a major target of F14512 in cultured cells, it is important to note that such a hypothesis would not be consistent with previous data indicating that cells deficient for Topo II are less sensitive to F14512 than Topo II-proficient cells [24], [25], hence strengthening the conclusion that Topo II plays a direct role in the cytotoxic action of the drug.

A global approach to a search for chromosomal cleavage F14512-induced Topo II cleavage sites reveals that TopoII is a pivotal component of drug activity. Our results show that F14512 is indeed capable of inducing cleavage at discrete sites in the *Drosophila* genome (Figure 4). In this case, we were able to document the dose-dependent F14512-specific disappearance of 2 *Hin*dIII fragments, concomitant with the appearance of a third fragment that most likely corresponds to a cleavage product. Regardless of the exact identity of fragments a, b and c, their behavior is unique to F14512-treated cells and is not seen in control or etoposide-treated samples. We consider this last point to be particularly important as it serves to establish a significant difference between these compounds. Again, with the *caveat* that more work will be needed before firmer conclusions can be drawn, we propose that the spermine moiety grafted onto F14512, and absent from etoposide or other Topo II poisons, endows this new molecule with unique properties that target it to discrete sites in chromatin, a conclusion supported by the remarkable effects of F14512 in whole developing animals.

### Drug-induced chromatin remodeling and alteration of gene expression *in vivo*

A particularly detailed understanding of *Drosophila melanogaster* genetics, which extends over one century of research, renders this organism eminently suitable for classical biological studies but also, as demonstrated here, to search for insights into the mechanism of action of new anti-cancer drugs and to identify their molecular targets. Feeding *Drosophila* larvae different classes of drugs ranging from Topo II poisons to minor-groove binders as well as histone-deacetylase inhibitors was previously shown to result in gain-of-function phenotypes [39], [53], [60], [61], [62], [63]. Here, we have extended this approach to the study of the effects of F14512 treatment in developing larvae and of the ensuing phenotypes in surviving adults. Results of our experiments demonstrate effects that, in addition to being largely chromatin-mediated (Figure 7), also denote gains or losses of functions that affect specific and readily identifiable genes.

Feeding F14512 to mutant *white-mottled Drosophila* larvae leads to the striking *Eye wide shut* phenotype recovered at a high frequency of surviving adults (Figure 8). From 5 independent experiments starting with 30 L3 larvae in duplicate, we obtained 14 *Eye wide shut* individuals, all males that had undergone a homeotic transformation of the left eye into a first thoracic segment. Remarkably, the *Eye wide shut* phenotype corresponds exactly to that obtained following ectopic expression of the *Antennapedia* (*Antp*) gene in the eye imaginal disks of larvae [64]. *Antp* is a HOX-family gene involved in the differentiation of the thoracic and head segments of the fly. It is normally repressed in segments where its expression is stably extinguished by a Polycomb-group repression complex that, significantly, has been shown to require Topo II for its activity [43]. Hence, the transformation we obtain suggests that F14512 may promote aberrant Topo II activity at least at this locus and, in flies that survive treatment, is manifested in one eye in an exquisitely specific fashion. The accompanying unilateral disappearance of male sex combs on the same side as the transformed eye can similarly be correlated to a loss-of-function of the *Sex combs reduced* (*Scr*) gene. *Scr*, which also encodes a homeobox-containing transcription factor, lies within the *Antp* complex and is similarly subject to Polycomb-mediated regulation [65]. However, it is differentially affected by F14512 feeding, relative to the closely linked *Antp* gene.

The residual shrunken eye portion found within the anterior side of the transformed eye or the multiple small eyes seen after treatment with lower concentrations of F14512 (Figure 8C) also suggest a partial loss-of-function of the *eyeless* (*ey*) gene [66]. *ey* encodes the Drosophila ortholog of the PAX-6 transcription factor and is a “master control” gene for eye development [67]. *Antp* antagonizes its activity [64] and it is plausible that the *Eye wide shut* and shrunken-eye or small double-eye phenotypes both reflect *Antp* gain-of-function and concomitant *ey* loss-of-function. It should also be noted that *ey* is located on chromosome 4 [68], the smallest and most highly condensed of the 4 chromosome pairs present in *Drosophila melanogaster*. Chromosome 4 consists largely of heterochromatin, in which euchromatin regions are interspersed [69]. Alternatively, *ey* loss-of-function induced by F14512 might reflect a perturbation of the normal distribution of heterochromatin and associated proteins in the *white-mottled* line. In this respect, it is perhaps significant that we only recovered *Eye wide shut* males: the large Y chromosome of Drosophila consists almost entirely of heterochromatin and can perturb heterochromatin-mediated effects by titrating heterochromatin-associated proteins (reviewed in [70]).

The striking loss of symmetry that characterizes eye transformation and loss of sex combs is more difficult to explain. This could be due to a perturbation of signaling pathways and/or genes implicated in structural symmetry. The *Notch* (*N*) gene is required for the maintenance of certain aspects of lateral symmetry [51], [52] and, perhaps more importantly in this case, has been implicated in the establishment of the axis of mirror symmetry in the *Drosophila* eye. *Notch* lies within 338.5 kbp of *white* and is therefore also relocalized near X-chromosome heterochromatin in the *white-mottled* inversion (Figure 6A; [47], [71]). Accordingly, we were able to document a drug-independent asymmetric variegation of eye color in large *white-mottled* collections (Figure 6B), most likely reflecting a unique tendency for one-sided effects in this genetic background, where the characteristic eye-color phenotype is highly sensitive to Topo II localization, activity or poisoning thereof [39], [53]. We surmise that the *white-mottled* background might provide a highly sensitized context that permits the expression of phenotypes such as *Eye wide shut*. In this case, the identification of exquisitely sensitive targets is in line with what appears to be a limited subset of F14512-induced Topo II cleavage sites in tissue-culture cells (Figure 4; [24]) even though this drug behaves similarly to other Topo II poisons *in vitro* (Figure 2).

These complex *in vivo* results obtained in a whole developing organism —although they can be reconciled with known genetic anomalies— constitute a remarkable instance of specific alterations of gene expression by ingestion of a drug. F14512 is unique in this respect: the *Eye wide shut* phenotype was never recovered after feeding teniposide, m-amsa, etoposide or idarubicin (data not shown) to L3 *white-mottled* larvae. Similarly, feeding F14512 to wild-type Oregon R larvae gave rise to no observable phenotypes, other than death, at the highest concentrations tested. We consider it particularly significant that F14512 exhibits a range of activities not found in related drugs such as etoposide and teniposide, suggesting singular mechanisms of action consistent with unusual targeting of discrete Topo II cleavage sites. We hypothesize that F14512 effects might be promoted, at least in part, by facilitated uptake, *via* its polyamine moiety, by larvae and tissues therein [72].

### *Drosophila*-based anticancer pharmacology

Taken together, these results establish the validity of using a fully integrated *Drosophila melanogaster* system to study the mode of action of a new anticancer drug, tentatively identify its targets and to unravel its most specific aspects as obtained phenotypes can be easily linked to target genes. While not a substitute for experimentation in the mouse for example, which provides invaluable cancer and tumor xenograft models, *Drosophila* offers, as shown here, a powerful, rapid and inexpensive complement that could also integrate feeding of multiple drugs that might affect the effects of drugs such as F14512 and their polyamine moieties and could thus serve as adjuvants. We expect that other genetic backgrounds would extend the suitability of this system to the analysis of different classes of drugs. As recently discussed, such a notion is indeed fully consistent with the outcome of recent elegant molecular studies that, due to its “genetic tractability,” firmly establish *Drosophila melanogaster* as a system of choice for the study of tumorogenesis and cancer therapy [73].

In conclusion, we have documented the usefulness of the *Drosophila* model system to characterize the properties of F14512, a novel targeted antitumor agent, and to distinguish it from epipodophyllotoxin-containing Topo II inhibitors. In addition to the presence of a spermine moiety which targets F14512 preferential entry into cancer cells —the rational basis for its initial design— our study, using a predisposed genetic background that is hypersensitive to Topo II dysfunction or mislocalization, identifies a unique subset of target genes. Such a property might of course be extremely useful to develop new drugs whose targets belong to a restricted subset, perhaps enhancing cytotoxic effects while limiting, in normal cells, the genome-wide damage that can result from the accumulation of frequent drug-induced DNA lesions. As such, F14512, which is currently being evaluated in a phase I clinical trial in patients with acute myeloid leukemia, is expected to exert promising anticancer activities.
